# Effects of Sand-Coated and Ribbed GFRP Bars in Hybrid GFRP-Steel-Reinforced Concrete Beams

**DOI:** 10.3390/ma19071372

**Published:** 2026-03-30

**Authors:** Rajeev Devaraj, Ayodele Olofinjana, Christophe Gerber

**Affiliations:** School of Science, Technology and Engineering, University of the Sunshine Coast, 90 Sippy Downs Dr., Sippy Downs, QLD 4556, Australia; aolofinj@usc.edu.au (A.O.); cgerber@usc.edu.au (C.G.)

**Keywords:** GFRP-concrete bond, hybrid GFRP-steel beams, sand-coated and ribbed surface profiles

## Abstract

The integration of glass fibre-reinforced polymer (GFRP) and steel reinforcement in hybrid RC beams offers durability benefits, yet the specific influence of GFRP surface treatments on bond mechanics remains critical. This study experimentally investigates the performance of hybrid GFRP-steel-reinforced beams under three-point bending, comparing sand-coated and ribbed GFRP bars, while maintaining a constant total reinforcement ratio of 1.4% to isolate interface mechanics. Due to the exploratory nature of the study and the specific specimen matrix, the results are interpreted as observed experimental trends rather than statistically generalised performance metrics. The results indicate that ribbed GFRP bars provide enhance mechanical interlocking; in this specific experimental program, the ribbed GFRP hybrid beam exhibits an observed load capacity approximately 11% greater than the sand-coated specimen in this study and surpassing comparable steel-only beams. Additionally, ribbed configurations demonstrated an observed 15% higher toughness. In contrast, sand-coated hybrid beams exhibited signs of premature bond degradation, quantitatively captured by strain gauge monitoring; sand-coated bars plateaued at 14,000 µε, reaching only 79% of their theoretical rupture capacity. This strain limitation indicates failure by internal slippage rather than material rupture, further evidenced by a 50% reduction in crack propagation compared to ribbed beams. While energy-based ductility indices suggest a marginal 6% advantage for sand-coated bars, both hybrid systems exhibited relatively low energy-based ductility indices (μ < 2), reflecting the linear-elastic nature of GFRP reinforcement. These findings suggest that the mechanical interlock of ribbed surface treatments is more resilient under the combined stress states typical of hybrid configurations, providing a foundational baseline for the development of future numerical models and reliability-based design frameworks for hybrid GFRP-steel-RC systems.

## 1. Introduction

The development of hybrid GFRP-steel-RC beams addresses key challenges in structural engineering, particularly in environments where corrosion resistance and durability are paramount. Steel reinforcement, widely known for its high ductility and energy-dissipating properties, faces significant limitations in aggressive environments due to corrosion-related durability issues. GFRP bars, on the other hand, provide superior durability and high tensile strength-to-weight ratios but lack the ductility and energy dissipation characteristics of steel. As a result, hybrid reinforcement systems, which combine steel and GFRP bars, offer a promising solution that leverages the strengths of both materials, using steel for ductility and energy dissipation, and GFRP for corrosion resistance and reduced maintenance requirements. To address the inherent complexities of FRP-reinforced concrete, research has increasingly focused on diverse strategies, ranging from reliability-based performance assessments [1] to the implementation of hybrid strengthening systems [2] aimed at optimizing material synergy. Previous studies [3,4,5] have demonstrated that hybrid GFRP-steel-RC beams can enhance structural performance, with potential applications in marine structures, bridge decks, and other high-durability requirements.

Central to the performance of hybrid RC beams is the bond behaviour of GFRP bars to concrete, which differs markedly from that of traditional steel reinforcement due to the nature of GFRP materials and their surface characteristics. Bond behaviour directly influences load transfer, crack control, and failure modes within RC elements, making it critical for the effective design and application of hybrid beams [6,7].

Recent investigations have further elucidated these complex bond mechanisms, highlighting the roles of confinement, fibre reinforcement, and concrete composition in optimising the stress transfer of FRP-RC members [8,9]. While these studies explore advanced concrete matrices, they underscore the universal importance of interface mechanics in defining structural ductility, a key focus of the present study on hybrid beam surface treatments.

Unlike steel bars, which have standardised ribbed surfaces for bond effectiveness, GFRP bars are available with various surface treatments, such as sand-coating or ribbing, to enhance bond interaction with concrete. Previous studies [10,11] suggest that these surface modifications significantly affect the bond-slip behaviour of GFRP bars, with important implications for load-sharing and crack distribution in hybrid RC beams. However, the comparative effectiveness of sand-coated versus ribbed GFRP bars in hybrid systems remains underexplored, especially in terms of their influence on key structural behaviours such as load-deflection response, energy dissipation, and ductility.

In this study, we investigate the effects of sand-coated and ribbed GFRP bars in hybrid GFRP-steel-reinforced beams, focusing on various behavioural and structural performance indicators. Specifically, we analyse the behaviour stages and failure process, crack patterns at failure, energy dissipation, secondary stiffness, ductility index, toughness, steel yielding moment, and moment-carrying capacity. By evaluating these parameters experimentally, this research aims to provide insights into the most effective GFRP surface treatments for hybrid beams and contribute to the development of more resilient and durable RC structures.

## 2. Background

Recent research has expanded the understanding of hybrid FRP-steel systems through both numerical and experimental investigations. Previous studies [12,13] have utilised numerical modeling to predict the flexural behaviour of hybrid beams in alternative concrete matrices, establishing benchmarks for theoretical validation. Furthermore, the critical influence of reinforcement ratios on strength and ductility has been extensively characterized [14], providing a framework for load capacity prediction in hybrid elements [15]. Within this broader context, the bond between GFRP bars and concrete is a critical factor influencing the structural performance of reinforced concrete elements, especially under load. Unlike steel bars, which achieve reliable bond performance due to ribbed surfaces and ductile properties, GFRP bars have varying bonding capabilities depending on their surface treatment. Sand-coated and ribbed GFRP bars represent two prominent surface types that affect bond strength differently. Many researchers have argued that sand-coating enhances the bond strength and reduces slip, and ribbed GFRP bars have relatively less bond strength. However, a recent study conducted by R. Devaraj et al. [16] concluded that, although sand-coated bars have slightly higher bond capacity, ribbed bars have better mechanical interlocking. The study also recommended assessing the performance of these bar types based on their global behaviour. Hence, comparison of these surfaces is significant in hybrid systems, where GFRP bond quality impacts composite action and load-sharing with steel.

In hybrid configurations where steel and GFRP bars are placed adjacently, the disparity in tensile properties creates a complex bond-stress environment. Due to its significantly higher modulus of elasticity (E_s_ ≈ 200 GPa) compared to GFRP (E_f_ ≈ 40–60 GPa), the steel reinforcement initially attracts the majority of the tensile force, effectively ‘shielding’ the GFRP bond during the elastic phase. However, this interaction changes drastically upon steel yielding. As the steel undergoes plastic deformation, the rapid increase in curvature forces a sudden stress transfer to the GFRP bars. At this critical juncture, the bond interface must be sufficiently robust to accommodate the surge in local slip; otherwise, the stiffness mismatch can trigger premature debonding, compromising the composite action of the hybrid section.

The load-deflection behaviour of hybrid beams reinforced with GFRP and steel typically progresses through distinct stages, each marked by changes in stiffness and load-bearing capacity. A previous study [17] has established that GFRP-reinforced beams, compared to steel-reinforced counterparts, undergo more abrupt stiffness changes due to the brittle nature of GFRP bars. The GFRP modifies failure mechanisms by delaying the steel yielding phase and influencing crack propagation patterns due to differences in ductility between materials. The progressive failure behaviour is dependent on the bond quality of GFRP, influencing the beam’s composite response and failure load [18,19].

Cracking patterns are another area where the choice of GFRP surface treatment can have a significant impact. Cracks in concrete beams typically initiate at lower loads and propagate more widely in GFRP-reinforced sections due to the lack of yielding compared to steel. Sand-coated bars, due to improved bond interaction, contribute to closer crack spacing and more extensive crack distribution, which is beneficial in controlling damage and postponing failure [20,21]. In contrast, ribbed GFRP bars exhibit evenly distributed cracks due to differing frictional mechanics [16,22]. This difference is essential for understanding failing hybrid beams, where the crack pattern can dictate the extent and sequence of steel yielding.

Hybrid GFRP-steel-reinforced concrete beams exhibit distinct energy dissipation characteristics resulting from the complementary behaviour of brittle GFRP and ductile steel reinforcement. Although GFRP bars inherently possess lower energy dissipation capacity compared to steel, the hybrid configuration significantly enhances overall ductility and ensures structural integrity under high cyclic loading conditions. Furthermore, among different types of GFRP bars, surface characteristics play a critical role in governing energy dissipation and influencing other key mechanical behaviours, such as bond performance and crack propagation.

Although various studies [21,23] have compared the characteristics of sand-coated and ribbed GFRP rebars, limited information is available on their behaviour within hybrid reinforcement systems. This represents a clear gap requiring investigation to enhance design optimisation and inform material selection and other critical design decisions.

## 3. Experimental Program

A total of three beams were produced for the experimental study: two hybrid GFRP-steel-RC beams and one fully steel-reinforced beam. In the hybrid configurations, one beam utilised sand-coated GFRP bars as primary reinforcement, while the other used ribbed GFRP bars. In both hybrid arrangements, steel reinforcement was placed internally, close to the primary longitudinal GFRP bars. The fully steel-reinforced beam had a greater reinforcement area compared to the hybrid beams, though this reduction in area in the hybrids was expected to be offset by the increased strength of the GFRP bars.

For the hybrid-reinforced beams, the reinforcement was arranged in two distinct layers. The analytical model calculates the unified effective depth (*d*) based on the area-weighted centroid of the steel and GFRP bars. Adopting the stress block parameters from AS3600:2018, the model accounts for the differing strain levels in each layer through the assumption of a linear strain distribution across the section height.

**Figure 1:**
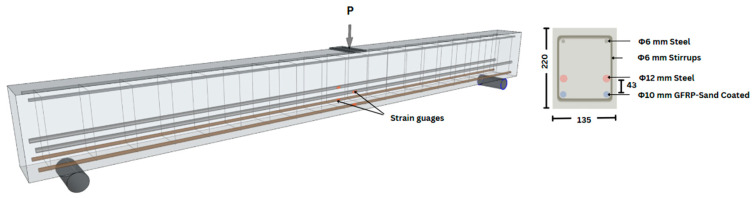
Test Beam and Reinforcement Configuration.

Each beam measured 2100 mm in length, with a cross-sectional dimension of 135 mm in width and 220 mm in depth. The primary GFRP reinforcement was placed at an effective depth of 198 mm, while the steel bar was positioned at an effective depth of 155 mm. Shear reinforcement consisted of 6 mm diameter steel stirrups spaced at 150 mm, which were secured directly to the bottom longitudinal reinforcement layer to maintain consistent effective depth, supported by two stirrup hangers at the top. Each beam was tested to failure under a three-point flexure loading, following the configuration and dimensions shown in Figure 1. The test constraints result in the experimental beam having a lower shear span to depth ratio, which enables a combined shear and flexure failure. However, this constraint is not expected to significantly impact the observations or the primary scope of the study.

The shear span-to-depth ratio of this study was approximately 4.8, calculated based on a shear span of 950 mm and an effective depth of 198 mm. While this falls within the range typically associated with combined shear–flexure behaviour, it remains above the threshold of 2.5 commonly used to define deep beam action. Nevertheless, the influence of this configuration on the reported results warrants explicit consideration. In FRP-reinforced systems, a reduced shear span elevates the diagonal compression field and increases bond demand along the longitudinal reinforcement in the shear span, particularly near the supports where force transfer between bar and concrete is most critical [24,25]. Unlike steel bars, which benefit from mechanical dowel action, GFRP bars rely primarily on surface-dependent bond mechanisms, making them more sensitive to the elevated interfacial shear demands associated with lower a/d ratios. Consequently, the bond degradation observed in the sand-coated specimens, evidenced by strain plateau at 79% of rupture capacity, may reflect a combination of flexural bond slip and shear-zone bond demand, rather than purely flexure-driven interface failure. This interaction cannot be fully decoupled within the present experimental design and represents a limitation in the mechanistic interpretation of bond-related observations.

The experimental program was designed as a comparative evaluation of two distinct GFRP surface treatments within a hybrid reinforcement configuration. While single specimens were utilised for each configuration (*n* = 1), the primary objective was to observe relative differences in global beam behaviour, such as load capacity, secondary stiffness, and crack distribution associated with the surface treatment type under identical loading and geometric conditions. The quantitative results are therefore presented as specific experimental observations indicative of how different GFRP surface treatments may influence overall flexural response, rather than as statistically generalised performance conclusions. These observations are intended to serve as indicative benchmarks for future replicated investigations and numerical model development.

### 3.1. Materials

The concrete used to cast the beam was supplied from a commercial ready-mix plant, confirming a mix of Portland cement with fine sand and 10 mm coarse aggregate for an average compressive strength of 40 MPa.

**Figure 2:**
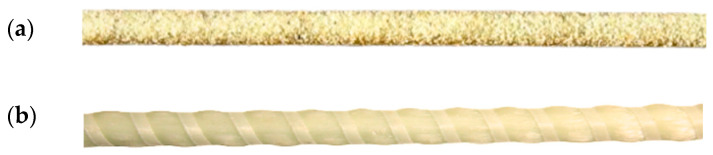
GFRP Bars. (**a**) Sand-coated; (**b**) Ribbed.

Reinforcement bars with a 10 mm diameter (Figure 2) were used as primary reinforcements. The product was manufactured to satisfy the specifications of ACI 440.5R-08. As with most of the commercially available GFRP bars, the GFRP bar used in this study was also manufactured by the protrusion method using E-glass as a primary fibre material and coating it with epoxy resin.

A commercially available deformed bar of Grade 500 steel with a diameter of 12 mm was used along with the GFRP bar as a tensile zone reinforcement. A 6 mm round steel bar conforming to AS/NZS 3679.1 Grade 300 was used as shear and hanger reinforcement. Table 1 shows reinforcement details.

A direct comparison between steel-reinforced and GFRP-reinforced concrete beams based on equal reinforcement areas may have limited accuracy due to the significant differences in their mechanical properties, most notably tensile strength and elastic modulus. For this reason, the present study focused on using a hybrid GFRP–steel reinforcement design that delivers optimal structural performance while enabling a meaningful comparison with a steel-only control beam possessing a comparable, or slightly greater, total reinforcement area. Consistent with the reinforcement arrangement from previous investigations, the use of relatively small-diameter GFRP bars in hybrid configurations is expected to promote ductile response governed by steel yielding rather than premature concrete crushing or GFRP rupture.

### 3.2. Preparation of Test Specimens

**Figure 3:**
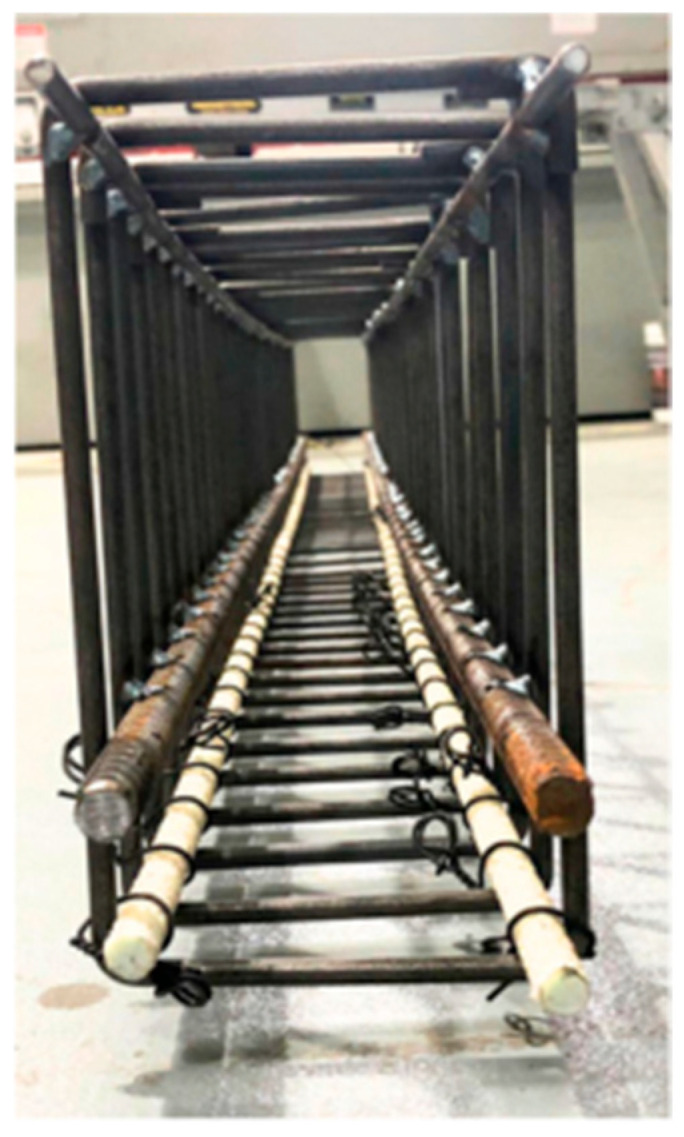
Reinforcement Cage.

**Figure 4:**
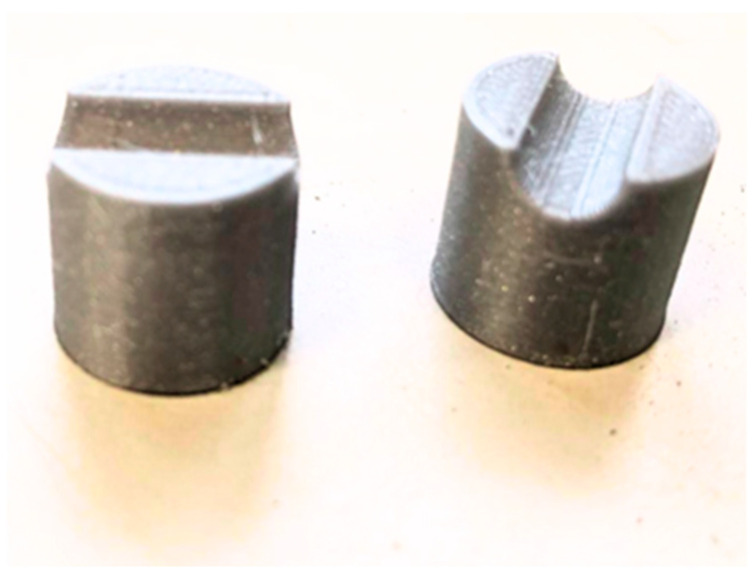
3D-Printed Reinforcement Chairs.

**Figure 5:**
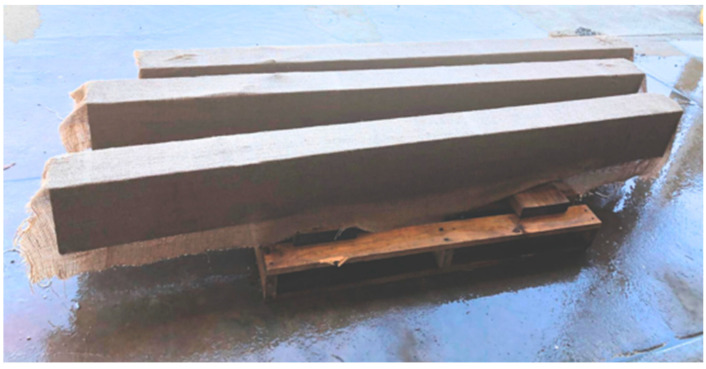
Beam curing under wet cloth.

Thick plywood boards were used to set up the mould for beam specimens. A thin layer of grease was applied to internal surfaces to make the unmoulding process easy and to resist water absorption. The steel longitudinal bars and hanger bars were tack-welded to the R6 stirrups while GFRP reinforcements were tied to the stirrup caging using steel wires, as shown in the cross-sectional view in Figure 3. To ensure the cover thickness as designed, plastic reinforcement chairs (Figure 4) were 3D printed and clipped to the stirrups. While GFRP stirrups offer enhanced corrosion resistance, steel stirrups were utilized in this experimental phase to ensure sufficient shear capacity and to isolate the flexural bond performance and mechanical interlocking behaviour of the longitudinal GFRP bars as the primary variables. The concrete was prepared at a ready-mix plant and transported to the laboratory. Thorough compaction was ensured while pouring each successive layer of concrete into the beam mould. The concrete beam remained in the mould for 3 days, afterward, it was taken out by unmoulding the form work and covering it with a wet cloth for curing, as shown in Figure 5. Three 100 × 200 mm cylinders were cast to determine the compressive strength of concrete, and they were cured under the same conditions as the beam specimen.

### 3.3. Test Setup and Procedure

**Figure 6:**
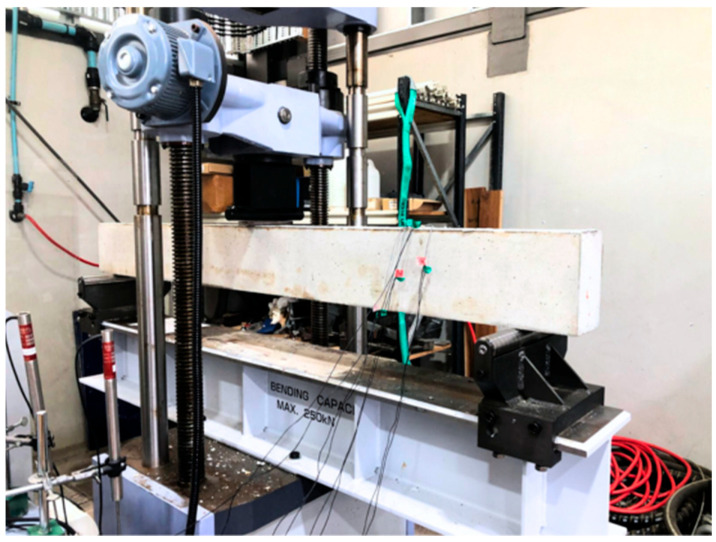
UTM loaded with the test beam.

A universal testing machine (UTM) of 600 kN capacity was used to facilitate the flexure testing. The test beam was loaded under three-point loading along a clear span of 1900 mm, as shown in Figure 6. A rubber membrane was placed at the contact point between the loading plate and the concrete surface to facilitate the efficient distribution of load over the surface. The speed of load application was set to be 1 mm/min, and the UTM’s loading head displacement was recorded to measure the deflection of the test beam. The data recorder was also used simultaneously to capture the readings from strain gauges bonded at the midspan of primary rebars. Specifically, 3 mm electrical resistance strain gauges (120 Ω resistance) were installed on the prepared surface of the longitudinal steel and GFRP bars at the mid-span location to monitor tensile strains.

## 4. Results and Discussion

### 4.1. Behaviour Stages and Failure Process

**Figure 7:**
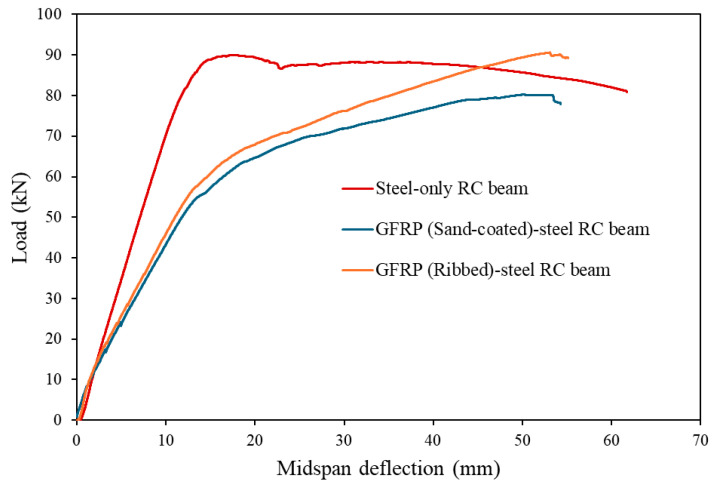
Load-deflection curves of tested beams.

The load-deflection curves for all tested hybrid GFRP-steel-reinforced concrete (RC) beams, shown in Figure 7, illustrate a load-deflection behaviour. Initially, the beam exhibits elastic behaviour due to chemical and mechanical adhesion between the materials within the RC beam, resulting in a linear curve. Notably, the steel-only control beam exhibited a steeper slope (higher stiffness) in this phase compared to the hybrid specimens. This is attributed to the significantly higher modulus of elasticity of steel (200 GPa) compared to GFRP (47–59 GPa), which results in a greater effective flexural rigidity for the steel-reinforced section. Furtherly, minor flexure cracks begin to appear in the concrete as chemical adhesion weakens, causing a reduction in stiffness. At this point, deflections were measured to be between 1 and 1.2 mm, approximately 2% of the peak deflection, as shown in Table 2. In the next stage of failure, occurring after concrete cracking but before steel yielding, shows an almost linear curve as the beam maintains stable stiffness. Deflections in this stage represent about 25–27% of the peak deflection.

After the steel bar yields, the stiffness of the beam continues to decrease, leading to rapid increases in mid-span deflection. Despite this, the load-deflection curve remains nearly linear, indicating stable stiffness up to the point where either the GFRP bar or the outermost compressive concrete fibre reaches its strain limit, at which point, failure is likely to begin. Due to its lower rupture limit compared to steel, the GFRP bar is expected to rupture first, initiating the collapse of the beam. Finally, the failure stage occurs once the peak load is reached. This stage is marked by the descending branch of the load-deflection curve, as the beam undergoes either GFRP rupture or concrete crushing, leading to complete failure.

A comparison between the hybrid sand-coated GFRP- and ribbed steel-RC beams shows that both follow similar behavioural patterns across these stages. However, the hybrid GFRP ribbed-steel beam demonstrated an observed load-carrying capacity approximately 11% higher than the sand-coated beam in this single-specimen comparison. This observed difference, while indicative, should be interpreted as a trend supported by mechanistic evidence rather than a statistically validated conclusion. Although the sand-coated and ribbed GFRP types have similar cross-sectional areas and mechanical properties, the difference in their surface characteristics significantly impacted load-carrying capacity in the hybrid reinforcement arrangement.

### 4.2. Crack Patterns at Failure

**Figure 8:**
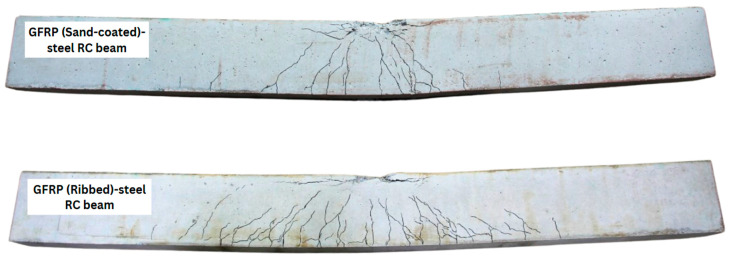
Crack patterns on tested beams.

Crack patterns were observed and marked following the failure of the tested beams, as shown in Figure 8, with real-time monitoring of crack formation conducted during testing. The crack distribution on both beams was nearly symmetrical, extending over a length of 900 mm on the hybrid RC beam with sand-coated bars and 1200 mm on the beam with ribbed GFRP bars. Results indicate that the hybrid RC beam with sand-coated bars exhibited relatively fewer cracks, whereas the beam with ribbed bars developed more than twice as many major and minor cracks. This difference points to the bond behaviour of the longitudinal bars, particularly the primary reinforcement. The higher mechanical resistance provided by the ribbed bars led to an increased number of cracks. While sand-coated bars typically offer continuous bonding, the specimens in this study experienced internal slippage due to coating degradation. In contrast, the ribs provided superior mechanical interlocking that resisted interfacial shear more effectively, promoting sustained stress transfer and crack propagation. In contrast, the hybrid RC beam with sand-coated bars exhibited fewer cracks, likely due to internal slippage.

The strain gauge readings provide further quantitative evidence of internal slippage in the sand-coated GFRP beams. The measured strain in the primary reinforcement flattened at approximately 14,000 µε before failure. Given the specific bar properties, the theoretical ultimate strain is calculated to be 17,660 µε. This disparity, where the bar reached only 79% of its rupture capacity, indicates that the limiting factor was not the tensile strength of the material but rather the degradation of the bond interface, which prevented further stress development.

It is also important to acknowledge the potential influence of the shear span-to-depth ratio on the observed crack patterns. At a/d ≈ 4.8, the shear span introduces an oblique stress component that can modify crack inclination, spacing, and propagation sequence relative to a purely flexure-dominated configuration. The higher crack density observed in the ribbed GFRP beam may therefore reflect not only superior mechanical interlocking but also the interaction between enhanced bond stiffness and the diagonal tension field present in the shear span. Similarly, the reduced crack propagation length in the sand-coated beam, while primarily attributed to internal slippage, may have been further influenced by the altered stress distribution in the combined shear-flexure zone. These effects cannot be independently quantified without companion tests at higher a/d ratios, and this remains a boundary condition on the mechanistic conclusions drawn from crack pattern analysis.

This observation aligns with the findings of R. Devaraj et al. [16] on bond behaviour of GFRP bars, where specimens with ribbed bars typically failed by concrete splitting, whereas those with sand-coated bars mostly failed by internal slippage, as the epoxy coating tended to peel off.

### 4.3. Ductility Index and Secondary Stiffness

**Figure 9:**
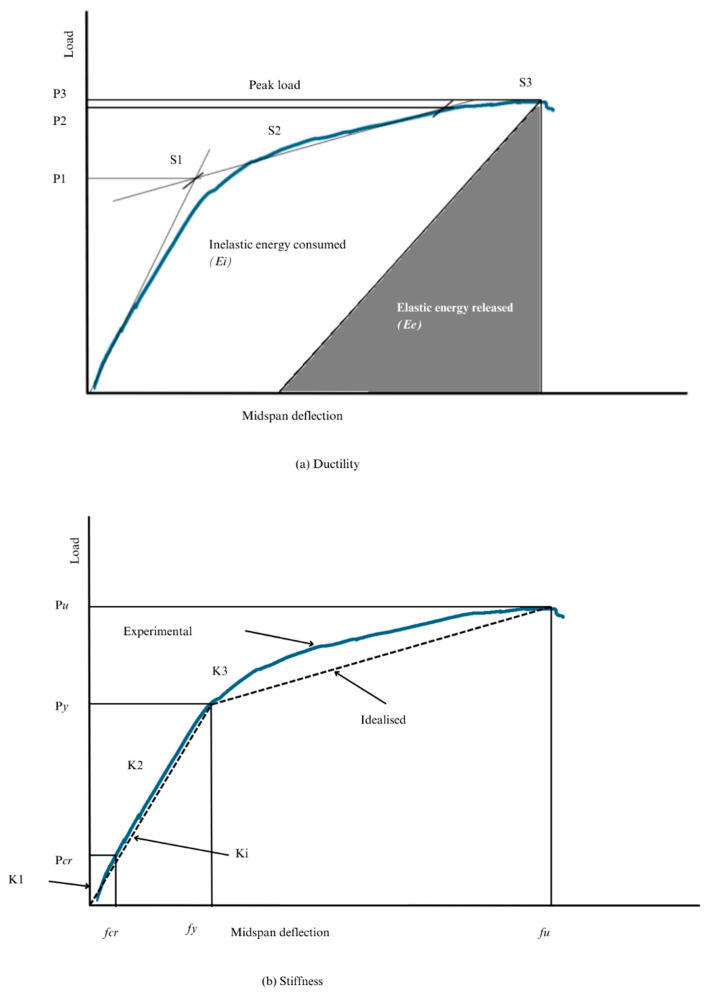
Ductility and stiffness index curves.

In hybrid GFRP-steel-reinforced concrete beams, ductility refers to the ability of the beam to undergo large deformations before failure, primarily contributed by steel, which allows energy dissipation and load redistribution during high-stress events. Similarly, stiffness is the resistance of a beam to deformation under load, and benefits from both materials: GFRP offers high tensile strength but lower stiffness, while steel compensates by increasing rigidity, especially in tension zones. In this study, considering that GFRP bars do not have yielding behaviour, an energy-based approach was adopted to determine the ductility indices of the hybrid RC beam. It is calculated using the ratio of the total energy of the load-deflection curve divided by the elastic energy as per Equation (1). Figure 9 presents the idealised load-deflection diagrams illustrating the process used to determine the ductility and stiffness of the tested hybrid RC beams. In Figure 9a, Stage S1 (Pre-yielding) corresponds to the point in the loading cycle immediately prior to the yielding of the longitudinal steel reinforcement. Stage S2 (Peak Strength) represents the state at the maximum load-carrying capacity achieved by the hybrid system. Finally, Stage S3 (Ultimate/Failure) denotes the state at which the beam reached its ultimate strain limit or experienced final failure.

(1) $$\mu =\frac{1}{2}\left(\frac{E_{t}}{E_{e}}+1\right)$$

where *E_t_* and *E_e_* refer to total energy and elastic energy released at failure, respectively. *E_t_* can be computed using Equation (2):

(2) $$E_{t}=E_{i}+E_{e}$$

According to Figure 9a, the slope of the line dividing the elastic energy (*E_e_*) from the inelastic energy (*E_i_*) can be obtained as follows:

(3) $$S=\;\frac{P_{1}S_{1}+\left(P_{2}-P_{1}\right)S_{2}}{P_{2}}$$

An alternative approach for determining the ductility indices of RC beams is to use the deformability concept (μ). This method considers the ultimate moment and curvature in relation to the moment and curvature at the concrete compressive strain, providing a measure of the deformability of the RC beam. However, conventional displacement ductility does not account for the secondary stiffness, which can lead to an overestimation of ductility in hybrid RC beams. While the current study includes an analysis of displacement ductility, it is used only for comparison purposes, as summarised in Table 3.

Additionally, the initial equivalent stiffness, *K_i_*, considered before steel yielding, and the secondary stiffness, *K*_3_, considered after steel yielding of the hybrid GFRP-steel-RC beams, were both calculated and analysed. The calculated stiffness values derived from Figure 9b can be defined as follows:

(4) Ki=Pyfy

(5) $$K_{3}=\frac{P_{p}-P_{y}}{f_{p}-f_{y}}$$

where *P_y_*, *f_y_*, and *P_p_*, *f_p_* are listed in Table 3.

For the evaluation of the structural parameters for hybrid GFRP-RC beams, specific deformation limits were established: stiffness was characterised using the deflections corresponding to the cracking point and yielding point; ductility was determined based on the transition at the yield point; and toughness was calculated by integrating the load-deflection response up to the ultimate deflection.

The energy-based ductility indices in Table 3 indicate that the hybrid RC beam with sand-coated GFRP exhibits about 6% greater ductility than the one with ribbed GFRP rebar. This increase reflects the higher bond-strength capacity of sand-coated GFRP bars, consistent with prior findings [16]. Generally, improved ductility is associated with a well-bonded composite interface that efficiently transfers stresses and promotes gradual failure of the beam after reaching ultimate load. However, in sand-coated GFRP bars, bond deformation is influenced by the strength of the epoxy layer, potentially causing sudden failure if the epoxy layer internally fails. This is evident in the load-deflection curve in Figure 7, where the sand-coated GFRP bar shows a sharp drop after reaching its peak load, highlighting this phenomenon.

A comparison between the definitions reveals a critical discrepancy in safety classification. As shown in Table 3, the conventional Displacement Ductility (µ∆) yielded values of 3.66 for sand-coated and 4.02 for ribbed hybrid beams. According to standard RC design limits, these values (>3.0) would incorrectly classify the beams as ‘ductile.’

However, the Energy-based Ductility (µ) provided significantly lower values of 1.90 and 1.78, respectively, correctly capturing the brittle nature of the failure mode. This divergence occurs because displacement ductility relies on yield displacement definitions that are ambiguous in hybrid sections with secondary stiffness, leading to an overestimation of deformability. Consequently, the energy-based approach is deemed more reliable for classifying the safety of hybrid GFRP-steel components.

Additionally, the ductility index (µ) was evaluated to compare the energy absorption characteristics of the different reinforcement configurations. While the thresholds of µ > 3 and µ < 2 are traditionally established for conventional steel-reinforced members, they are adopted here as provisional indicators to facilitate a relative performance comparison. In hybrid systems, ductility is a manifestation of both the plastic yielding of steel and the sustained deformability provided by the GFRP bars. The results indicate that the ribbed hybrid specimen approaches the ductile threshold more closely than the sand-coated counterpart, primarily due to the enhanced bond integrity, which allows for more extensive crack distribution and larger deformations prior to concrete crushing. However, these indices should be viewed as indicative of relative toughness rather than absolute design limits for hybrid members.

To classify the tested beams by their ductility, the energy ratio *E_i_*/*E_e_*, defined as the ratio of inelastic energy to total energy, was calculated. It is acknowledged that ductility classification thresholds conventionally used in steel-RC design, such as μ > 3 for ductile behaviour and μ < 2 for brittle behaviour, originate from frameworks that assume yielding reinforcement and are not directly transferable to hybrid FRP-steel systems where the FRP component behaves linearly elastic until rupture. Consequently, rather than applying these thresholds as definitive classification criteria, the energy-based ductility indices are presented here purely as relative performance indicators to facilitate comparison between the two hybrid configurations. In this context, the marginally higher ductility index of the sand-coated hybrid beam (μ = 1.90) compared to the ribbed hybrid beam (μ = 1.78) suggests a modest difference in energy absorption characteristics between the two surface treatments, consistent with the higher bond-strength capacity of sand-coated bars noted in prior investigations [16]. The steel-only beam is included for reference purposes, recognising that its ductility response is governed by fundamentally different mechanisms. The development of ductility classification frameworks specifically calibrated for hybrid FRP-steel-RC systems remains an open research need, and the indices reported here are intended to contribute comparative experimental data toward that goal. This behaviour is quantitatively explained by the reinforcement details provided in Table 1. Both hybrid beams were designed with a total reinforcement ratio of 1.4%, which is well below the calculated balanced ratio of 3.0%.

While this under-reinforced configuration typically encourages yielding, the low modulus of elasticity of the GFRP bars (47 GPa for sand-coated and 59 GPa for ribbed, as shown in Table 1) resulted in a delayed stiffness response compared to the steel-only beam. Consequently, while this constant ratio allowed for a direct comparison of surface treatments, it is evident that optimising the steel-to-GFRP area ratio is required to shift the hybrid system towards a fully ductile failure mode.

The internal mechanics of the hybrid system were evaluated by partitioning the tensile force between the steel and GFRP layers. As shown in Table 4, the steel reinforcement dominates the initial stiffness at Stage S1. However, upon steel yielding, a significant ‘force handover’ occurs. In the ribbed GFRP specimens, the GFRP contribution increased from 29% to 55% at the ultimate stage, whereas in the sand-coated specimens, this transition was less efficient due to observed bond-slip. This quantification confirms that the hybrid action relies on the GFRP’s ability to arrest cracks and sustain incremental loads once the steel capacity is exhausted.

The internal force distribution between the steel and GFRP reinforcement layers presented in Table 4 was determined through a strain-gauge-informed sectional analysis. The tensile force carried by each reinforcement layer at each critical loading stage was calculated using the measured midspan strains recorded by the electrical resistance strain gauges installed on the longitudinal steel and GFRP bars, in accordance with the following expressions:

(6) $$F_{s}=\varepsilon_{s}\cdot E_{s}\cdot A_{s}$$

(7) $$F_{f}=\varepsilon_{f}\cdot E_{f}\cdot A_{f}$$

where $\varepsilon_{s}$ and $\varepsilon_{f}$ are the measured tensile strains in the steel and GFRP bars, respectively, at each loading stage, $E_{s}$ and $E_{f}$ are the corresponding elastic moduli, as listed in Table 1, and $A_{s}$ and $A_{f}$ are the respective cross-sectional areas of the reinforcement. The loading stages S1, S2, and S3 correspond to the pre-yielding, peak strength, and ultimate states identified from the load-deflection response, with the associated applied loads listed in Table 4. It should be noted that beyond steel yielding (Stages S2 and S3), the steel force was taken as the product of the yield strain and elastic modulus, consistent with the onset of plastic deformation. The GFRP force at these stages was calculated directly from the measured strain as GFRP exhibits linear elastic behaviour up to rupture. This partitioning approach provides a first-order quantification of load-sharing evolution and is subject to the assumption of uniform strain distribution at the midspan cross-section.

### 4.4. Toughness

Toughness (*U_T,p_*) measures the ability of a beam to absorb energy and resist fracture throughout its deformation. Represented by the area under the load-deflection (or stress–strain) curve, it indicates the total energy the beam can endure before failure, which is crucial for structures subject to dynamic or impact loads as it reflects the capacity of the beam to deform and distribute stress gradually. The toughness of the tested beams in this study, calculated as the area under the load-deflection curve, is presented in Table 3.

The test results indicate that the hybrid RC beam with ribbed GFRP shows approximately 15% higher ductility than the beam with sand-coated GFRP rebar. This improvement is attributed to the bond characteristics of the ribbed GFRP bar, where the ribbed surface provided a robust mechanical interlock that maintained load transfer efficiency after primary adhesion between GFRP surface and concrete weakened. Unlike the sand-coated specimens, which suffered from premature bond degradation (slippage), the ribbed deformations facilitated sustained energy dissipation, reducing the risk of premature failure. This even stress distribution enhances the energy absorption capacity of the reinforcement bar before breaking. The ribs also increase surface area, increasing load transfer efficiency between the GFRP and the surrounding concrete, contributing to overall beam toughness. Additionally, ribbed GFRP bars dissipate more energy through friction and mechanical interlocking than smooth or sand-coated bars, a key factor in their improved toughness.

It is acknowledged that, given the single-specimen experimental design at the structural scale, this numerical difference represents an observed trend rather than a statistically confirmed finding. The mechanistic basis for this improvement, namely the superior mechanical interlocking of ribbed bars sustaining load transfer after adhesion loss, was independently established through the replicated pull-out study (*n* = 6) reported in [16].

### 4.5. Steel Yielding Moment and Moment-Carrying Capacity

The steel yielding load listed in Table 2 is determined by the load at the point of sudden change in deflection on the load–displacement curve shown in Figure 7. It can be observed that the hybrid RC beam with ribbed GFRP has a slightly higher yielding point of about 4% compared to the hybrid RC beam with sand-coated GFRP. This increase in yielding point can be attributed to the enhanced bond strength and mechanical interlock provided by the ribbed GFRP bars, which improve load transfer and stress distribution.

Additionally, the moment carrying capacity is also higher for the hybrid RC beam with ribbed GFRP, with about 11% improvement compared to the hybrid RC beam with sand-coated GFRP and about 1% improvement compared to the comparable steel-only RC beam. Among many additional factors, this increased moment-carrying capacity can be primarily explained by its strain hardening effect. The strain hardening effect increases the yield strength of the steel, which means it can sustain higher loads before yielding. This directly contributes to the higher yielding point observed in the hybrid RC beams with ribbed GFRP. Additionally, the increased yield strength enhances the overall moment-carrying capacity of the hybrid beams, as the steel reinforcement can carry higher loads without failing.

It should be noted that this performance comparison considers the efficiency of the reinforcement system rather than a direct material substitution. Although the hybrid beams utilized a lower total reinforcement area compared to the steel-only control beam, the superior tensile strength of the ribbed GFRP bars allowed the hybrid system to achieve a comparable or superior moment capacity.

## 5. Conclusions

This study presents a comparative experimental evaluation of sand-coated and ribbed GFRP bars in hybrid GFRP-steel-reinforced concrete beams tested under three-point bending. The following conclusions are drawn from single-specimen observations (*n* = 1) and should be interpreted as indicative experimental trends under the specific conditions tested, rather than as statistically generalised performance conclusions. The directional consistency of these observations with material-level bond behaviour established in prior replicated pull-out testing [16] lends mechanistic support to the trends reported.

- In this experimental program, ribbed GFRP bars in hybrid RC beams exhibited an observed 11% higher load and moment-carrying capacity compared to the sand-coated configuration. This observed difference is consistent with the superior mechanical interlocking of ribbed bars, which resisted premature bond slippage under the increased interface demand following steel yielding.
- Beams with sand-coated GFRP bars showed indicative signs of internal slippage, evidenced by a strain plateau at 79% of theoretical rupture capacity and a crack propagation length approximately 50% less than that observed in beams with ribbed GFRP bars. These observations suggest that bond interface degradation, rather than material rupture, was the governing limit state in the sand-coated configuration under the conditions of this study.
- Energy-based ductility indices indicated a marginal observed difference between the two configurations, with the sand-coated hybrid beam showing approximately 6% greater ductility than the ribbed counterpart. These indices are presented as relative comparative indicators rather than definitive classifications, as conventional steel-RC ductility thresholds are not directly applicable to hybrid FRP-steel systems where FRP reinforcement behaves linearly elastic until rupture.
- The ribbed GFRP hybrid beam exhibited an observed 15% higher toughness than the sand-coated configuration, reflecting the sustained energy dissipation facilitated by mechanical interlocking after primary adhesion weakened. This observed difference is presented as an indicative trend consistent with the constitutive behaviour of ribbed bars in prior material-level investigations.

These findings provide comparative observational evidence that GFRP surface treatment meaningfully influences structural-level performance indicators in hybrid RC beams under the conditions tested. However, it is acknowledged that the single-specimen experimental design limits the statistical generalisability of these observations. Future studies should investigate larger sample groups, varied reinforcement configurations, and higher shear span-to-depth ratios to statistically validate these trends and decouple shear-flexure interaction effects from the flexural bond behaviour under investigation. Furthermore, the quantitative observations reported here, including strain development, force partitioning ratios, and crack pattern data, are intended to serve as indicative benchmarks for the calibration of future numerical simulations and analytical models of hybrid FRP-steel-RC systems.

## Figures and Tables

**Table 1:** Details of reinforcement used in test beams.

Type of Beam	Elastic Modulus (GPa)			Area of Tensile Reinforcement (mm^2^)			Reinforcement Ratio (%)	Balanced Reinforcement Ratio (%)
	GFRP-SC	GFRP-Ribbed	Steel	GFRP-SC	GFRP-Ribbed	Steel		
GFRP (Sand-coated)-steel-RC beam	47	-	200	142	142	226	1.4	3.0
GFRP (Ribbed)-steel-RC beam	-	59	200	142	142	226	1.4	3.0
Steel-only RC beam	-	-	200	-	-	452.4	1.98	1.69

**Table 2:** Hybrid GFRP-steel-RC beam testing results.

Type of Beam	No. of Cracks		Cracking Point		Yielding Point			Peak Point			Ultimate Point
	Major	Minor	P*cr*, kN	∆*cr*, mm	∆*y*, kN	M*y*, kNm	∆*y*, mm	P*p*, kN	M*u*, kNm	∆*p*, mm	∆*u*, mm
GFRP (Sand-coated)-steel-RC beam	8	9	8.5	1.2	55.2	27.6	13.7	80.2	40.1	50.13	50.13
GFRP (Ribbed)-steel-RC beam	16	23	7.3	1.06	57.4	28.7	13.2	90.6	45.3	53.05	53.05
Steel-only RC beam	-	-	22.3	3.2	82.5	41.25	12.4	89.9	44.95	17.5	61.7

**Table 3:** Stiffness, ductility and toughness of tested beams.

Beam Type	Stiffness			Ductility						Toughness Ut,p, MPa
	K*_i_*, kN/mm	K_3_, kN/mm	r*b*	E*el*, kNmm	E*_i_*, kNmm	E*_i_*/E*_t_*, %	Relative Ductility	µ∆	µ	
Steel-only RC beam	6.65	1.45	0.21	525.87	4274.3	89.04	Reference	4.98	5.06	76.96
GFRP (Sand-coated)-steel-RC beam	4.03	0.69	0.17	1015.5	1828.8	64.30	Lower	3.66	1.9	45.61
GFRP (Ribbed)-steel-RC beam	4.35	0.83	0.19	1297.1	2030.5	61.02	Higher	4.02	1.78	53.35

**Table 4:** Internal force distribution and contribution ratios at critical stages.

Performance Stage	Load (kN)	Steel Force (F_*s*_, kN)	GFRP Force (F_*f*_, kN)
S1 (Pre-Yielding)	~30–35	110.4	45.2
S2 (Peak Strength)	~70–78	113.1	118.5
S3 (Ultimate)	~65–70	113.1	135.8

## Data Availability

The original contributions presented in this study are included in the article. Further inquiries can be directed to the corresponding author.
